# Characterization of *Streptococcus salivarius* as New Probiotics Derived From Human Breast Milk and Their Potential on Proliferative Inhibition of Liver and Breast Cancer Cells and Antioxidant Activity

**DOI:** 10.3389/fmicb.2021.797445

**Published:** 2021-12-15

**Authors:** Kantapich Srikham, Wichittra Daengprok, Piyanuch Niamsup, Mongkol Thirabunyanon

**Affiliations:** ^1^Program in Biotechnology, Faculty of Science, Maejo University, Chiang Mai, Thailand; ^2^Program in Food Science and Technology, Faculty of Engineering and Agro Industry, Maejo University, Chiang Mai, Thailand

**Keywords:** anticancer, antioxidant, breast milk, cancer, lactic acid bacteria (LAB), probiotics

## Abstract

Breast milk is well known as the abundant source of beneficial bacteria. A new alternative source of human probiotic origin from breast milk is in demand and currently of interest for both the functional food industry and biopharmaceuticals. The aim in this study was to investigate the anticancer and antioxidant efficacies of the new potential probiotics isolated from human breast milk. Three strains of lactic acid bacteria (LAB) have shown their potential probiotic criteria including antimicrobial activity, non-hemolytic property, and survival in acid and bile salt conditions. These strains showed high abilities on cell surface hydrophobicity, auto-aggregation, and co-aggregation. The genera identification by 16S rRNA sequencing and comparison revealed that they were *Streptococcus salivarius* BP8, *S. salivarius* BP156, and *S. salivarius* BP160. The inhibition of liver cancer cells (HepG2) and breast cancer cells (MCF-7) proliferation by these probiotic strains using a 3-(4,5-dimethylthiazol-2-yl)-2,5-diphenyltetrazolium bromide (MTT) assay was 44.83–59.65 and 29.85–37.16%, respectively. The probiotic action mode was inducted via apoptotic mechanisms since they stimulate the liver and breast cancer cell death through DNA fragmentation and positive morphological changes by acridine orange (AO) and propidium iodide (PI) staining. The antioxidant activity of these probiotics in the form of intact cells, cell free supernatant (CFS), and heat-killed cells was evaluated by a 2,2–diphenyl–1–picrylhydrazyl (DPPH) assay, resulting in the scavenging activity rates of 16.93–25.43, 15.47–28.03, and 13.67–23.0%, respectively. These *S. salivarius* probiotic strains protected the L929 mouse fibroblasts against oxidative stress with very high survival rates at 94.04–97.77%, which was significantly higher (*P* < 0.05) than L-ascorbic acid at 75.89–78.67% in the control groups. The results indicated that *S. salivarius* BP8 and *S. salivarius* BP160 probiotic strains could be applied as functional foods or new alternative bioprophylactics for treating liver and breast cancers.

## Introduction

Cancer disease is one of the main causes of human death worldwide. Breast cancer is the first leading cause of death in women and liver cancer is the second leading cause of death in men (Ferlay et al., 2019). At present, chemotherapy and radiation are the treatment for cancer. Since the therapeutic cost of these treatments is relatively high and the appearance of many side effects often occurs, new alternative bioprophylactics and/or biotherapy is now a necessity for treating cancers.

Probiotic bacteria have many beneficial functions in the gastrointestinal tract to improve human health (Sanders, 2003). Most of them are lactic acid bacteria (LAB) including genera *Lactobacillus, Enterococcus, Pediococcus*, and *Streptococcus* (Ringø et al., 2018; Yang et al., 2019). Probiotics have immunostimulatory abilities such as anti-inflammatory effects in the gastrointestinal tract (Plaza-Díaz et al., 2017) and inhibition of pathogens by metabolite production (Rushdy and Gomaa, 2013). Probiotic bacteria can be found and isolated from fermented foods (Lee et al., 2016), dairy products (Pisano et al., 2014), and in human sources, e.g., oral and saliva (Bosch et al., 2012), vagina (Nami et al., 2014), and infant feces (Tarrah et al., 2019).

For their applications, probiotics have been widely used in the food industry supplemented as functional food nutrients, fermented food products, dairy products, and beverages (Turkmen et al., 2019; Graham et al., 2020; Marx et al., 2020). In addition, probiotics are important in pharmaceuticals in terms of their immune enhancement properties (Putta et al., 2018). Currently, probiotics are a promising anticancer regimen since they are a new alternative for cancer treatment referred to as bioprophylactics and biotherapy. In earlier reports, probiotics acted against colon cancer (Thirabunyanon et al., 2009) and gastric cancer (Russo et al., 2007). Recent investigations of probiotics have expanded to other cancers such as liver and breast cancers (Luang-In et al., 2020; Jafari-Nasab et al., 2021). The potential mechanisms of probiotics on prevention and therapy have several action modes. They include inhibiting the growth of pathogenic bacteria, activating the apoptotic programmed cell death of cancer cells, and inducing the immune response (Górska et al., 2019).

The antioxidant activity of probiotics was also recently reported (Kim et al., 2020; Hoffmann et al., 2021). It is known that probiotic metabolites are effective antioxidants by expressing scavenging activity against 2,2–diphenyl–1–picrylhydrazyl (DPPH) and hydroxyl free radical. Moreover, the intact cell and heat-killed cell of probiotics are also effective antioxidants (Jang et al., 2018; Son et al., 2018). Thus, probiotic consumption comprising antioxidant activity is capable of exerting more benefits to human health for protection against cancer and several diseases.

Human milk is well known to include the nutritional requirements of growing infants and an abundance of many beneficial bacteria (Fernández et al., 2013). Bacterial diversity of breast milk includes various genera such as *Lactobacillus*, *Lactococcus*, and *Streptococcus*, etc. (Martín et al., 2007). Also, breast milk bacterial efficacies promote infant health and stimulate the immune system (Perez-Cano et al., 2010; Fernández et al., 2013).

Thus, this study evaluated the probiotic characteristics of LAB isolated from human milk, its action modes, antioxidant efficacies, and the bioprophylactic strategy of these probiotic strains against liver and breast cancers.

## Materials and Methods

### Lactic Acid Bacterial Isolation

Human breast milk samples were donated by healthy lactating women from the hospitals of Chiang Mai, Thailand. Milk samples were serially diluted and spread on MRS agar plates (Criterion, United States). Different colonies were randomly selected and streaked onto the MRS agar. After incubation, a single colony of each isolate was subjected to Gram staining to evaluate the morphology. Finally, each isolate was stored at –20°C for further experiments.

### Antimicrobial Activity

The inhibitory activity of LAB isolates was determined as previously described (Seddik et al., 2017). Antimicrobial property of LAB isolates was tested against pathogenic bacteria, i.e., *Helicobacter pylori* DMST 20165, *Escherichia coli* TISTR 780, *Salmonella* Enteritidis DMST 15676, *Salmonella* Typhimurium TISTR 292, *Staphylococcus aureus* TISTR 118, *Bacillus cereus* TISTR 687, and *Listeria monocytogenes* DMST 1783. For this test, LAB isolates were cultured overnight in MRS broth (Criterion, United States) and 3 μl of each isolate was added on MRS agar plates. After 18 h, anaerobic incubation at 37°C was performed. Agar plates were overlaid with BHI soft agar (Criterion, United States) containing 0.5% (v/v) pathogenic strains and incubated at 37°C for 24 h. Clear zone diameters were measured using a vernier caliper.

The modified methods of Mohammadi et al. (2018) were adapted for the well diffusion assay.

All pathogen strains were cultured in BHI broth for 18 h. Then, the cultured pathogen strains were mixed with BHI agar at 45–50°C (0.05% v/v) and poured onto sterile petri dishes (Corning, United States). Wells were formed using a cork borer (0.6 mm diameter) and added with 10 μl of 1.5% agar. A total of 50 μl of LAB isolates cell free supernatant (CFS, pH 7.0) was pipetted into each well. Hydrogen peroxide (Merck, Germany) (0.3% v/v) was used as control. After 24-h incubation at 37°C, the inhibition zone was measured.

### Hemolytic Activity

The hemolytic activity of LAB isolates was confirmed according to Peres et al. (2014). LAB isolates were streaked on Columbia agar plates supplemented with 5% (v/v) human blood and incubated at 37°C for 48 h. After that, hemolytic characteristics, i.e., β-hemolysis, α-hemolysis, and γ-hemolysis were evaluated. The LAB strains that expressed γ-hemolysis were indicated as non-hemolytic and selected for further experiments.

### Acid and Bile Salt Tolerance Activity

LAB were tested for their ability to survive in the gastrointestinal tract environment via acid (Haghshenas et al., 2015) and bile salt tolerance assays (Oh and Jung, 2015; Son et al., 2018). To evaluate acid tolerance, LAB strains were cultured overnight in MRS broth, and 1 ml of cultured LAB was added to 9 ml of PBS solution and adjusted to pH 2.5 with HCl (5 N) (Merck, Germany). After incubation at 37°C, bacterial cell suspensions were serially diluted and spread on MRS agar. After 24 h of plate incubation, the surviving bacterial cells were measured at 0 and 3 h of incubation. Results were determined as log cfu/ml.

Likewise, bile salt tolerance was also tested with cultured LAB in MRS broth for 18 h and then 1 ml of the LAB strains was added to 9 ml of MRS broth supplemented with 0.3% of bile salt (w/v) (Sigma, United States). After incubation at 37°C, bacterial cell suspensions were serially diluted, spread on MRS agar, and incubated at 37°C. Bacterial cell survival was evaluated in MRS agar plates at 0 and 24 h. Results of this experiment were represented as log cfu/ml.

### Cell Surface Hydrophobicity

Based on bacterial adhesion to hydrocarbons, this assessment was performed following the method of Lee et al. (2017) with some modifications. The LAB strains were cultured overnight and cell pellets were collected by centrifugation (4,000 *g* for 10 min at 4°C). LAB cells were washed twice with PBS, resuspended in PBS, and adjusted to an absorbance of 1.0 at 600 nm (OD_A_). Then, 3 ml of bacterial suspensions was separately mixed with 600 μl of three hydrocarbons, i.e., n-hexadecane (Fluka, Germany), toluene, and xylene (Fisher, England). Tubes were vortexed for 30 s and kept at room temperature for 30 min. The aqueous phase was collected and absorbance at 600 nm (OD_B_) was measured. Results were expressed as percentage. Cell surface hydrophobicity was calculated by the following equation:


Cellsurfacehydrophobicity(%)=[1-(OD/BOD)A]×100(1)

### Auto-Aggregation and Co-aggregation

For auto-aggregation and co-aggregation, the method of Campana et al. (2017) with some modifications was performed. The LAB isolates were cultured in MRS broth. After 18 h, the pellet cells were washed twice with PBS and cell concentration was adjusted to 10^8^ cells/ml. Then, 4 ml of cell suspensions was mixed vigorously and incubated at 37°C for 6 h. Absorbance of OD_600_ was detected, and auto-aggregation (%) was calculated as follows:


Auto-aggregation(%)=[1-(A/6A)0]×100  (2)

Where A_0_ and A_6_ are the absorbance at 0 and 6 h, respectively.

For co-aggregation, LAB cell suspensions and pathogenic cell suspensions were prepared. Then, 4 ml of mixed cell suspensions (2 ml of each LAB cell suspension and 2 ml of each pathogenic cell suspension) were incubated at 37°C for 6 h. The absorbance of OD_600_ was measured, and co-aggregation (%) was calculated as follows:


Co-aggregation⁢(%)⁢=⁢[(Ax⁢+⁢Ay)/2–A(x⁢+⁢y)]/[Ax⁢+⁢Ay/2]  (3)

Where A*_x_* and A*_y_* are the individual absorbance of the LAB strain and pathogen, respectively, and A(*x* + *y*) is the absorbance of the LAB strain and pathogen in combination.

### Probiotic Identification

This assessment was performed following the method of Thirabunyanon and Thongwittaya (2012) with modifications. A single colony of each isolate was inoculated in MRS broth overnight, and pellet cells were separated by centrifugation. Genomic DNA from the pellet cells of each strain was extracted using a genomic DNA mini kit (Tiangen, Taiwan) following the manufacturer’s instructions. The genomic DNA was observed using 1.5% agarose gel electrophoresis. For 16S rRNA sequencing, amplification of the 16S rRNA region was conducted using a pair of universal primers: 27F and 1522R (Operon, Germany). The PCR program cycles were set as follows: denaturation at 95°C for 5 min; 25 cycles at 95°C for 1 min, 55°C for 1 min, and 72°C for 1 min; and final extension at 72°C for 5 min. The amplified PCR products were examined using 1.5% agarose gel electrophoresis and purified with a Tianquick midi purification kit (Tiangen, Taiwan). Then, the amplified 16S rRNA fragments were sequenced. Finally, the sequences were compared with the BLAST program of the National Center for Biotechnology Information (NCBI) to identify the genus and species of the probiotic strains.

### Antiproliferation of Liver and Breast Cancer Cells

#### Cell-Free Supernatant Preparation

The CFSs of probiotic bacteria were prepared. A single colony of each strain was cultivated in MRS broth and incubated at 37°C for 18 h in an anaerobic condition. Then, the OD of these incubated samples were adjusted to 0.800 ± 0.05. A total of 2 ml of each sample was added to 8 ml of MRS broth and incubated anaerobically at 37°C for 18 h. The active bacterial suspension was separated by centrifugation at 4,000 *g* at 4°C for 10 min and supernatants were sterilized by filtration through syringe filter units (0.22 μm). Consequently, the filtered cell-free supernatant was later used in antiproliferation and apoptotic assessment of cancer cells and the DPPH assay.

#### Cancer Cell Lines and Growth Condition

Human liver cancer cells (HepG2) and human breast cancer cells (MCF-7) obtained from the American Type Culture Collection (ATCC) were used in this study. Two cancer cell lines were routinely cultured in Dulbecco’s Modified Eagle Medium (DMEM, Gibco, United States) supplemented with 10% fetal bovine serum (FBS, Gibco, United States), 1% non-essential amino acid (Gibco, United States), and 1% penicillin-streptomycin (Gibco, United States). Cells were cultured in a 25 cm^3^ flask (Nunc, Denmark) and incubated in a CO_2_ incubator (Forma Scientific, 3111, United States) at 37°C under a 5% CO_2_ atmosphere and 95% humidity. These cell lines were later used for antiproliferation and apoptotic assessment of cancer cells.

#### MTT Assay

This assay was conducted by 3-(4,5-dimethylthiazol-2-yl)-2,5-diphenyltetrazolium bromide (MTT) following the modified method of Thirabunyanon et al. (2009). Briefly, 100 μl of HepG2 and MCF-7 cells (10^5^ cells/ml) was seeded onto the 96-well plate (Nunc, Denmark). After 24 h incubation, 100 μl of CFS was added to each well and MRS medium was used as control. All treated cells were incubated for 24 h. After incubation, 10 μl of MTT (Sigma, United States) solution (0.5 mg/ml in DMSO) was added to each well and plates were incubated in a dark condition for 4 h in 5% CO_2_ at 37°C. Then, 100 μl of DMSO (Sigma, United States) was added to each microplate well to fix the cells for 15 min at 37°C. The absorbance was read at 595 nm using a microplate reader. The growth inhibition rates were expressed as percentages calculated with the following formula:


%Antiproliferation=100×[1-(OD/s⁢a⁢m⁢p⁢l⁢eOD)c⁢o⁢n⁢t⁢r⁢o⁢l]  (4)

### DNA Fragmentation

This test was evaluated according to Salmanzadeh et al. (2018) with some modifications.

A total of 1 μl of either HepG2 or MCF-7 cells (10^6^ cells/ml) was seeded in a 24-well microplate.

After 24 h, 1 ml of CFS was added to each well and incubated for about 24 or 48 h. The supernatants and non-adhesive cells were discarded, and the adhesive cells were harvested by trypsinization. Genomic DNA of cancer cells was extracted using a DNA extraction kit (Tiangen, Taiwan). The extracted DNA was observed for DNA fragmentation on 1.5% agarose gel using gel electrophoresis.

### Apoptotic Cell Morphological Assessment

Acridine orange (AO; Sigma, United States) and propidium iodide (PI; Sigma, United States) staining was performed for morphological assessment (Hajiaghaalipour et al., 2015). In total, 500 μl of either HepG2 or MCF-7 cell suspension (10^6^ cells/ml) was cultured in an 8-well Chamber Slide system (Nunc, Denmark) for 24 h and treated with 500 μl of probiotic CFS. After 24 h of incubation, the medium was discarded; cancer cell morphological observation was performed by acridine orange (AO, 10 μg/ml) and propidium iodide (PI, 10 μg/ml) staining. Morphological assessment of fresh-stained cells was investigated under fluorescence microscopy (Olympus, Japan) within 30 min.

### Antioxidant Activity

#### Intact Cells and Heat-Killed Cell Preparation

Probiotic strains were cultured in MRS broth for 18 h. Intact cell pellets were washed twice and adjusted to 10^8^ cells/ml with PBS solution. For heat-killed cell preparation, the resulting cell suspension was sterilized for 15 min at 121°C by an autoclave. Both intact and heat-killed cell suspensions were later used for the DPPH assay.

#### DPPH Assay

The DPPH free radical scavenging activity of probiotic strains was assessed according to the modified method of Son et al. (2018). In brief, 3 ml of each sample, i.e., LAB CFS, intact cells, and heat-killed cells was separately added to 3 ml of methanolic DPPH solution (0.4 mM). The mixture was vortexed and incubated in a dark condition at 37°C for 30 min before analysis. Deionized water and MRS broth with DPPH solution were used as controls. After incubation, the solution was centrifuged at 8,000 *g* for 10 min and absorbance of supernatants was determined at 517 nm. The scavenging activity was estimated using the following equation:


Scavengingactivity(%)=100×[1-(OD/s⁢a⁢m⁢p⁢l⁢eOD)c⁢o⁢n⁢t⁢r⁢o⁢l](5)

### Protective Activity of *Streptococcus salivarius* Probiotics on Viability of Oxidation-Induced Cells

The protective activity of *S. salivarius* probiotics against the induction of oxidative stress was assessed following the modified method as previously described (Coda et al., 2012). The mouse fibroblasts (L929, ATCC) were cultured in DMEM supplemented with 10% FBS, 1% non-essential amino acid, and 1% penicillin-streptomycin (all from Gibco). L929 cells were sub-cultured in 25 cm^3^ flasks and incubated in a humidified 5% CO_2_ incubator at 37°C. Cell viability was measured using the MTT method which followed the modified method of Thirabunyanon et al. (2009).

For the cell oxidative stress assay, 100 μl of L929 cells (10^4^ cells/ml) was seeded onto the 96-well plates and incubated for 16 h. After incubation, 100 μl of CFS was added to each well and further incubated for 16 h. In this assay, L-ascorbic acid (Sigma, United States) was used as positive control. Subsequently, the cells were exposed to 20 μl (150 mM) of hydrogen peroxide for 2 h. After incubation, 10 μl of MTT solution (0.5 mg/ml in DMSO) was added to each well and plates were incubated in a dark condition for 4 h in 5% CO_2_ at 37°C. Finally, 100 μl of DMSO was added to solubilize the formazan. The absorbance was read at 595 nm using a microplate reader (Spectrostar Nano, Germany). Data were showed as percentage of viable cells of non-induced and oxidative stress-induced cells. Samples were conducted in triplicate and three independent experiments were performed.

### Statistical Analysis

Each sample was carried out in triplicate and assays were repeated at least twice. The experimental data were analyzed using SPSS version 25.0 software. Statistical differences between multiple groups were analyzed by one-way ANOVA and Duncan’s multiple range test. All numerical data were demonstrated as mean ± standard deviation (SD) and *P* < 0.05 indicated statistical significance.

## Results

### Characterization of Lactic Acid Bacteria Strains and Hemolytic Activity

After Gram staining of the strains, the morphology of all LAB strains was Gram-positive and spherical shaped. Also, these strains showed non-catalase production. Virulence investigation of LAB was further studied. The γ-hemolysis was expressed in the three LAB strains indicating non-hemolytic genera (data not shown).

### Antimicrobial Activity

The growth inhibition of foodborne pathogens by LAB strains is shown in Table 1. Although the LAB isolates BP8, BP156, and BP160 had activity against all pathogens, they did not differ among them. Inhibition zones against pathogenic *H. pylori* DMST 20165 were 11 mm, *E. coli* TISTR 780 were 10–11 mm, *S.* Enteritidis DMST 15676 were 14–16 mm, *S.* Typhimurium TISTR 292 were 13–15 mm, *S. aureus* TISTR 118 were 11 mm, *B. cereus* TISTR 687 were 13–14 mm, and *L. monocytogenes* DMST 1783 were 13–14 mm. After neutralization of supernatants and the well diffusion assay, results showed that all LAB isolates had no inhibitory properties against the growth of all tested foodborne pathogens (Table 1).

**TABLE 1:** Inhibition zone diameters (mm) of LAB strains isolated from human breast milk for seven different foodborne pathogens (mean ± SD, *n* = 3).

Bacterial strains	Spot on lawn assay			Well diffusion assay (CFS, pH 7.0)		
	BP8	BP156	BP160	BP8	BP156	BP160
*Escherichia coli* TISTR 780	11 ± 0	10 ± 1	11 ± 1	–	–	–
*Salmonella* Enteritidis DMST 15676	16 ± 1	16 ± 1	14 ± 2	–	–	–
*Salmonella* Typhimurium TISTR 292	15 ± 3	13 ± 1	14 ± 1	–	–	–
*Bacillus cereus* TISTR 687	14 ± 1	13 ± 1	13 ± 1	–	–	–
*Helicobacter pylori* DMST 20165	11 ± 1	11 ± 1	11 ± 1	–	–	–
*Listeria monocytogenes* DMST 1783	14 ± 1	13 ± 1	13 ± 3	–	–	–
*Staphylococcus aureus* TISTR 118	11 ± 1	11 ± 1	11 ± 1	–	–	–

*(–) No inhibition.*

### Acid and Bile Tolerance Activity

**FIGURE 1:**
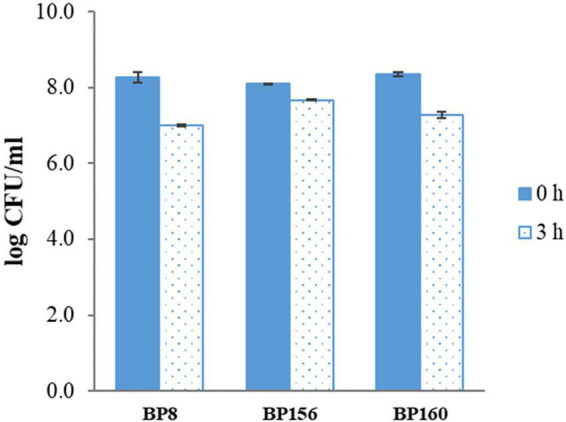
The acid tol erance activity of LAB isolates after 3-h incubation at pH 2.5 (means ± SD). Error bars indicate the standard deviation from three independent experiments.

**FIGURE 2:**
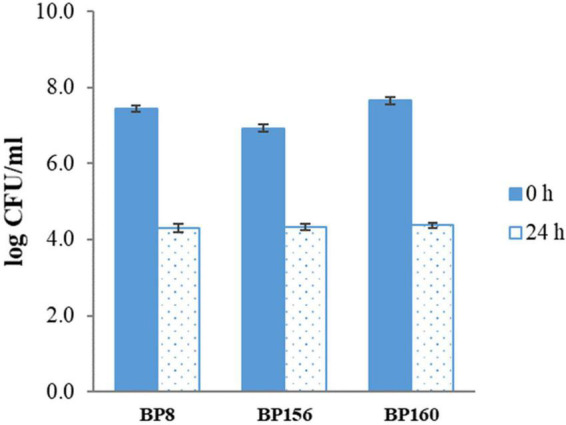
The bile salt tolerance activity of LAB isolates after incubation with 0.3% bile salts for 24 h (means ± SD). Error bars indicate the standard deviation from three independent experiments.

The tolerance to the gastric condition of BP8, BP156, and BP160 strains exposed to pH 2.5 is shown in Figure 1. The mild reduction of survival rates after 3 h of challenge was found in all isolates. Acid tolerance activity of BP8, BP156, and BP160 was reduced from 8.27 to 7.01, 8.10 to 7.68, and 8.35 to 7.68 log cfu/ml, respectively. After challenging the intestinal condition with 0.3% bile salt for 24 h, the reduction of survival rates of BP8, BP156, and BP160 were reduced from 7.44 to 4.30, 6.93 to 4.33, and 7.66 to 4.38 log cfu/ml, respectively (Figure 2).

### Cell Surface Hydrophobicity

**FIGURE 3:**
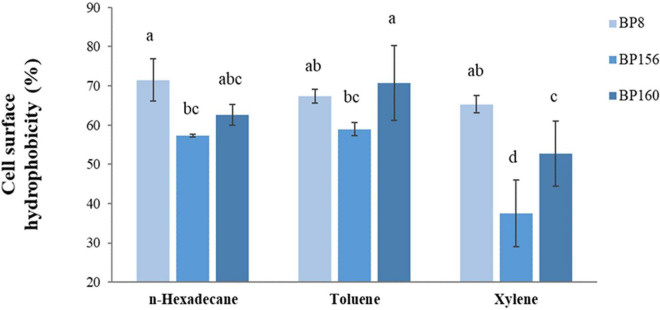
Cell surface hydrophobicity of LAB isolates (means ± SD). The letters above the bars denote significant difference (*P* < 0.05) as determined by Duncan’s multiple range test. Error bars indicate the standard deviation from three independent experiments.

The adhesive property of LAB strains was evaluated by cell surface hydrophobicity using n-hexadecane, toluene, and xylene. As shown in Figure 3, these strains exhibited high adhesion rates in which the hydrophobicity of *S. salivarius* strains was 57.34–71.46% with n-hexadecane, 58.89–70.76% with toluene, and lastly 37.52–65.31% with xylene, respectively.

### Aggregation Ability

The auto-aggregation and co-aggregation of the strains are shown in Table 2.

**TABLE 2:** Auto-aggregation and co-aggregation ability of LAB strains (mean ± SD, *n* = 3).

Probiotic strains	Auto-aggregation (%)	Co-aggregation (%)	
		*E. coli*	*S.* Enteritidis
*S. salivarius* BP8	27.13 ± 1.13	20.07 ± 0.92	20.82 ± 1.15
*S. salivarius* BP156	26.77 ± 2.19	19.14 ± 2.06	21.66 ± 2.00
*S. salivarius* BP160	26.37 ± 2.24	20.03 ± 1.44	21.19 ± 1.13

The auto-aggregation ability of *S. salivarius* strains was 26.37–27.13%. Also, co-aggregation efficacy of *S. salivarius* strains challenged with *E. coli* had the high rates of 19.14–20.07%, whereas *S.* Enteritidis challenged with *S. salivarius* strains resulted in high co-aggregation rates of 20. 82–21.66%.

### Probiotic Identification

Molecular identification of the BP8, BP156, and BP160 strains by the 16S rRNA gene was sequenced and compared to the GenBank. The genera were identified and defined as *Streptococcus salivarius* BP8 (accession no: KX246843.1), *S. salivarius* BP156 (accession no: NR_042776.1), and *S. salivarius* BP160 (accession no: MN400227.1) with an identity accuracy of 99.0, 99.2, and 99.9%, respectively.

### Proliferative Inhibition of Liver and Breast Cancer Cells

**FIGURE 4:**
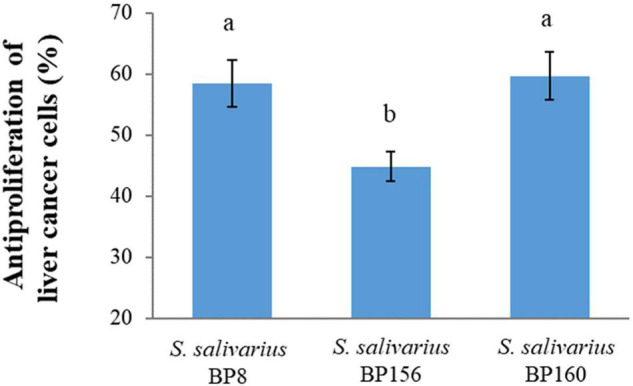
Antiproliferation activity of *S. salivarius* strains in HepG2 cell lines (means ± SD). The letters above the bars denote significant difference (*P* < 0.05) as determined by Duncan’s multiple range test. Error bars indicate the standard deviation from three independent experiments.

**FIGURE 5:**
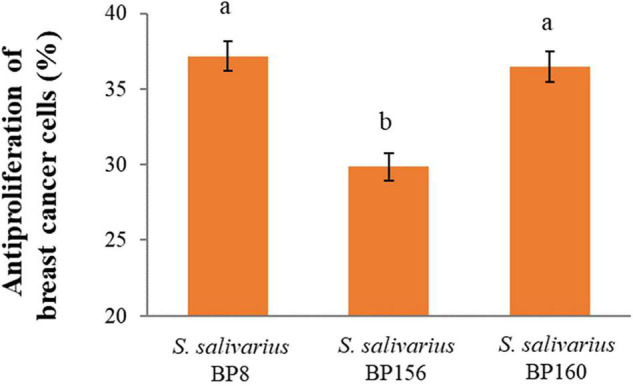
Antiproliferation activity of *S. salivarius* strains in MCF-7 cell lines (means ± SD). The letters above the bars denote significant difference (*P* < 0.05) as determined by Duncan’s multiple range test. Error bars indicate the standard deviation from three independent experiments.

The antiproliferative activity of probiotics by CFS on liver and breast cancer cells was assessed using an MTT assay. As shown in Figure 4, *S. salivarius* BP8, *S. salivarius* BP156, and *S. salivarius* BP160 had high inhibiting rates against liver cancer cell proliferation at 58.42, 44.83, and 59.65%, respectively. Moreover, probiotics *S. salivarius* BP8, *S. salivarius* BP156, and *S. salivarius* BP160 exhibited the ability to inhibit the growth of breast cancer cells at the rates of 37.16, 29.85, and 36.47%, respectively (Figure 5).

### DNA Fragmentation

**FIGURE 6:**
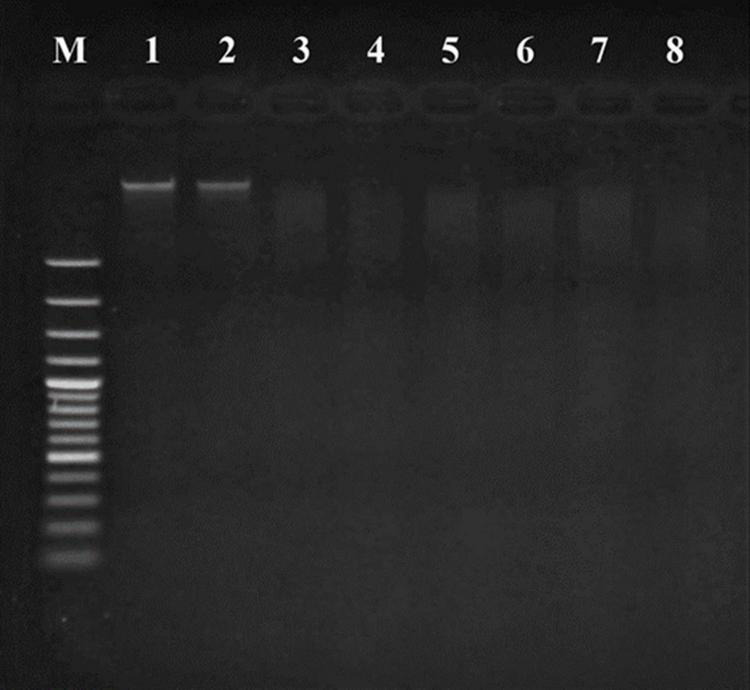
DNA fragmentation of HepG2 cell lines treated with CFS of *S. salivarius* strains for 24 and 48 h. MRS medium used as control (Lane M; DNA ladder, lane 1; control 24 h, lane 2; control 48 h, lane 3; BP8 24 h, lane 4; BP8 48 h, lane 5; BP156 24 h, lane 6; BP156 48 h, lane 7; BP160 24 h, and lane 8; BP160 48 h).

**FIGURE 7:**
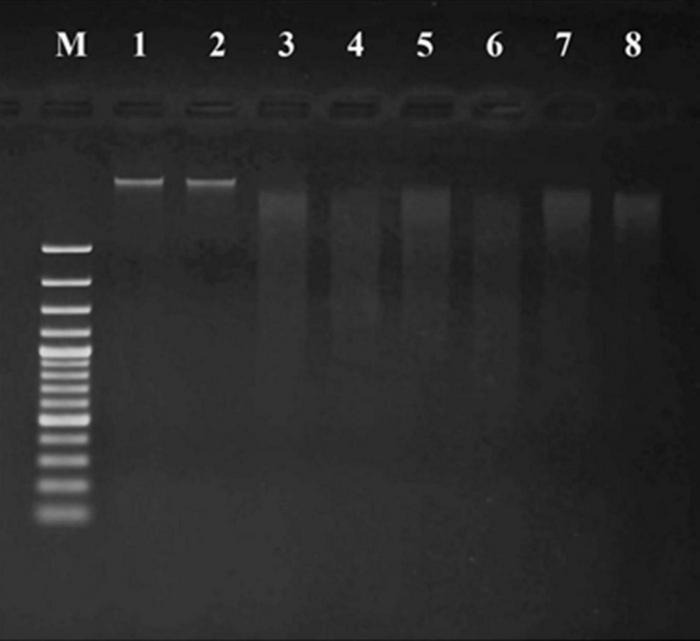
DNA fragmentation of MCF-7 cell lines treated with CFS of *S. salivarius* strains for 24 and 48 h. MRS medium used as control (lane M: DNA ladder, lane 1: control 24 h, lane 2: control 48 h, lane 3: BP8 24 h, lane 4: BP8 48 h, lane 5: BP156 24 h, lane 6: BP156 48 h, lane 7: BP160 24 h, and lane 8: BP160 48 h).

The apoptotic incidence in cancer cells treated with probiotics was observed through DNA fragmentation. The results represented both DNA bands of liver cancer cells (Figure 6) and breast cancer cells (Figure 7). Non-fragmented DNA was displayed in the control group of liver cancer cells treated with MRS medium for 24 h (lane 1) and 48 h (lane 2). Small (24 h treatment) and smaller (48 h treatment) DNA fragments of liver cancer cells treated with CFS of *S. salivarius* BP8 (lane 3; 24 h and lane 4; 48 h), *S. salivarius* BP156 (lane 5; 24 h and lane 6; 48 h), and *S. salivarius* BP160 (lane 7; 24 h and lane 8; 48 h) were, respectively, shown.

Non-fragmented DNA was observed in the control group of breast cancer cells treated with MRS medium for 24 h (lane 1) and 48 h (lane 2). After being treated with probiotics, the small (24 h treatment) and smaller (48 h treatment) DNA fragments of breast cancer cells with *S. salivarius* BP8 (lane 3; 24 h and lane 4; 48 h), *S. salivarius* BP156 (lane 5; 24 h and lane 6; 48 h), and *S. salivarius* BP160 (lane 7; 24 h and lane 8; 48 h) were, respectively, displayed.

### Morphological Change of Apoptotic Cancer Cells

**FIGURE 8:**
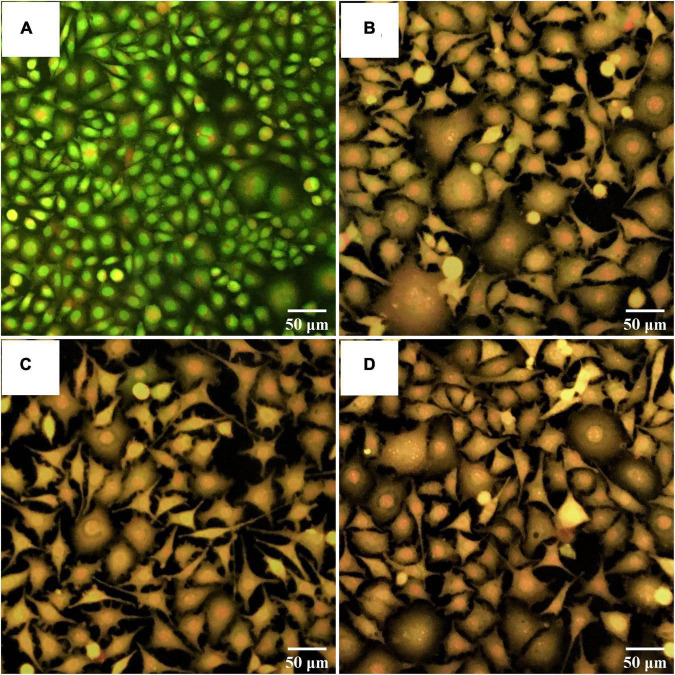
Fluorescent photomicrographs of AO/PI-stained cells on HepG2 cell lines treated with CFS of *S. salivarius* strains for 24 h. **(A)** Untreated cells, **(B)** HepG2 cells treated with *S. salivarius* BP8, **(C)** HepG2 cells treated with *S. salivarius* BP156, and **(D)** HepG2 cells treated with *S. salivarius* BP160.

An apoptotic induction by potential probiotics was investigated by AO/PI staining. The morphological change of non-treated liver cancer cells (control) is shown in Figure 8A with bright green stain indicating no apoptosis. In contrast, the morphological change of liver cancer cells treated with probiotics *S. salivarius* BP8 (Figure 8B), *S. salivarius* BP156 (Figure 8C), and *S. salivarius* BP160 (Figure 8D) was, respectively, expressed with orange stain indicating late apoptosis or death of cells.

**FIGURE 9:**
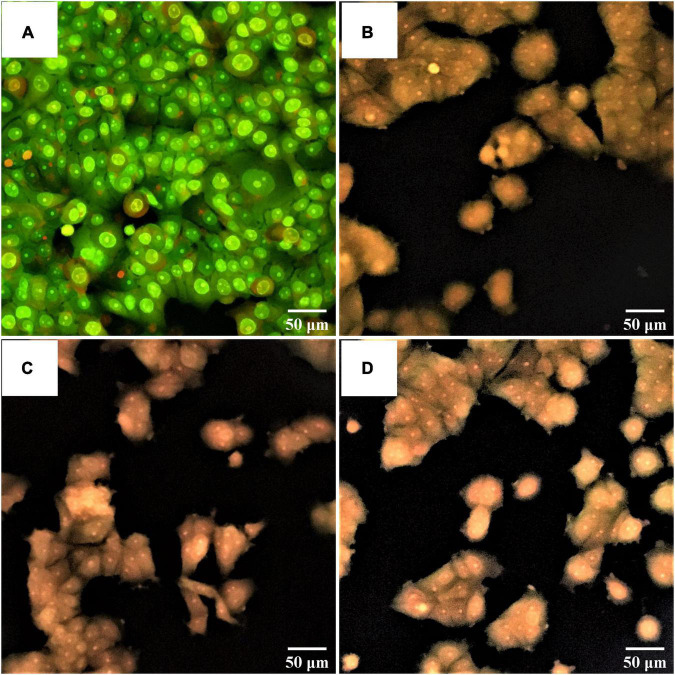
Fluorescent photomicrographs of AO/PI-stained cells on MCF-7 cell lines treated with CFS of *S. salivarius* strains for 24 h. **(A)** Untreated cells, **(B)** MCF-7 cells treated with *S. salivarius* BP8, **(C)** MCF-7 cells treated with *S. salivarius* BP156, and **(D)** MCF-7 cells treated with *S. salivarius* BP160.

The changes in cells with bright green of non-treated breast cancer cells (control) occurred, indicating no apoptosis (Figure 9A). Treated breast cancer cells with *S. salivarius* BP8 (Figure 9B), *S. salivarius* BP156 (Figure 9C), and *S. salivarius* BP160 (Figure 9D) resulted in an orange color that indicated late apoptotic or dead cells.

### Antioxidant Activity

**FIGURE 10:**
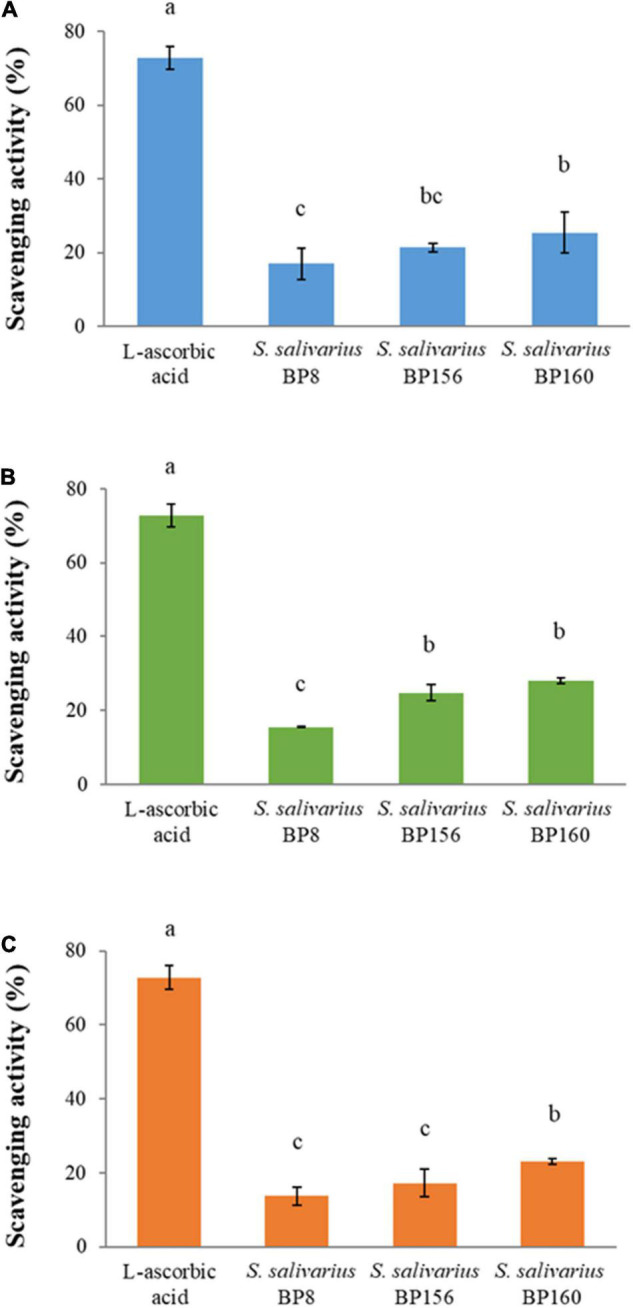
The scavenging activity of *S. salivarius* strains (means ± SD). L-ascorbic acid (100 μg/ml) was used as control. Bar graph **(A)** intact cells of *S. salivarius* strains, **(B)** CFS of *S. salivarius* strains, and **(C)** heat-killed cells of *S. salivarius* strains. The letters above the bars denote significant difference (*P* < 0.05) as determined by Duncan’s multiple range test. Error bars indicate the standard deviation from three independent experiments.

The scavenging activity of probiotics assessed by DPPH assay is shown in Figure 10. The scavenging activity of intact cells (Figure 10A), CFS (Figure 10B), and heat-killed cells (Figure 10C) was observed in different inducing forms of probiotics. Results showed that all of three probiotic forms had antioxidant activity in correlating constancy among probiotic strains, rating in higher to lower antioxidant activity from *S. salivarius* BP160, *S. salivarius* BP156, and *S. salivarius* BP8, respectively. However, the scavenging activity of these probiotics was significantly lower (*P* < 0.05) than the control (L-ascorbic acid). The scavenging activity of control, *S. salivarius* BP8, *S. salivarius* BP156, and *S. salivarius* BP160 in the probiotic forms of intact cells was 72.81, 16.93, 21.38, and 25.43%, CFS was 72.81, 15.47, 24.71, and 28.03%, and heat-killed cell was 72.81, 13.67, 17.22, and 23.0%, respectively.

### Protective Activity of *Streptococcus salivarius* Probiotics on the Viability of Oxidation-Induced Cells

**FIGURE 11:**
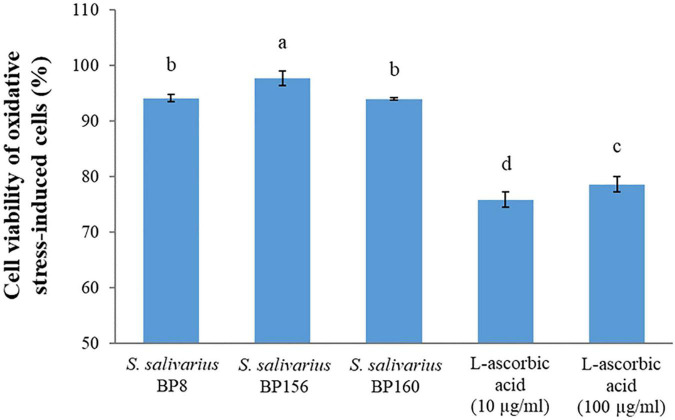
Protection activity of *S. salivarius* probiotics on the viability of L929 mouse fibroblasts after oxidative stress induction with hydrogen peroxide. L-ascorbic acid (10 and 100 μg/ml) was used as control. The letters above the bars denote significant difference (*P* < 0.05) as determined by Duncan’s multiple range test. Error bars indicate the standard deviation from three independent experiments.

The capacity of the *S. salivarius* probiotics to act as radical scavengers of cultured mouse fibroblasts after oxidative stress induction on cells is shown in Figure 11. After oxidative stress induction with hydrogen peroxide, these *S. salivarius* probiotic strains protected the cells against oxidative stress, resulting in very high survival percentages. As compared to the control groups (L-ascorbic acid), all these probiotic strains had higher and significant (*P* < 0.05) survival rates. The mouse fibroblast survival rates of *S. salivarius* BP8, *S. salivarius* BP156, *S. salivarius* BP160, L-ascorbic acid 10 μg/ml, and L-ascorbic acid 100 μg/ml were 94.17, 97.77, 94.04, 75.89, and 78.67%, respectively.

## Discussion

Probiotic bacteria are considered to be health beneficial bacteria when consumed in adequate amounts. Sources of probiotics include fermented foods (Rao et al., 2015), dairy products (Karami et al., 2017), and the gastrointestinal tract of human and animals (Musikasang et al., 2009; Thirabunyanon and Hongwittayakorn, 2013). Importantly, the human origin of probiotics has been focused on for potential applications. Probiotics originating from humans such as breast milk are accepted as beneficial to health (Fernández et al., 2013; Nagpal et al., 2018) since probiotic potential and anticancer functionalities of lactic acid bacteria isolated from human milk have been reported (Reis et al., 2016).

The present study found three bacterial strains, i.e., BP8, BP156, and BP160 derived from human breast milk and analyzed their probiotic properties and anticancer and antioxidant activities. Primarily, probiotic criteria of the strains were evaluated and justified. All of these strains had an antagonistic effect against seven pathogenic bacteria including *H. pylori* DMST 20165, *E. coli* TISTR 780, *S.* Enteritidis DMST 15676, *S.* Typhimurium TISTR 292, *S. aureus* TISTR 118, *B. cereus* TISTR 687, and *L. monocytogenes* DMST 1783. These findings indicate that they have potential as beneficial probiotics. The action modes of these probiotic strains could be from the antimicrobial substance production which includes lactic acid and hydrogen peroxide (Šušković et al., 2010). However, in this study, it might not be from bacteriocins or antimicrobial peptides. Pathogenic bacteria, such as *E. coli* are effective at producing carcinogens that induce colon cancer while *H. pylori* is also involved in inducing gastric carcinoma (Hatakeyama, 2009; Bonnet et al., 2014). Also, it has been reported that probiotic strain *Lactobacillus gasseri*, isolated from human milk, exhibited inhibitory effects on foodborne pathogens such as *E. coli*, *S. aureus, B. cereus*, and *S.* Enteritidis (Gunyakti and Asan-Ozusaglam, 2019).

The safety of probiotics is one important criterion for probiotic selection. Red blood cell hemolysis is the safety evaluation of probiotic bacteria adapted from “The 2002 FAO/WHO Guidelines on Probiotics Safety Considerations” (FAO/WHO, 2002). In this study, all strains, i.e., BP8, BP156, and BP160 were confirmed as non-hemolytic with γ-hemolytic observation. The γ-hemolytic activity of probiotic bacteria obtained from human milk has also been recorded (Gunyakti and Asan-Ozusaglam, 2019).

Acid and bile salt tolerances are two main important criteria for probiotics to be considered as human probiotics (Saarela et al., 2000). These aspects are applied to investigate if the probiotic could persistently tolerate extreme conditions in the human gastrointestinal tract and later exert functional proprieties. In this study, the survival ability was evaluated through exposure in the simulated gastrointestinal tract of humans. Accordingly, the tolerance activity of BP8, BP156, and BP160 was found. Similarly, a previous study reported that *Lactobacillus* isolates from human breast milk in the simulated human gastrointestinal (GI) tract had a survival rate of over 46–65% (Jamyuang et al., 2019). Moreover, the tolerance activity of probiotic *Lactobacillus* and *Pediococcus* was also reported. These probiotics could resist pH of 2.5 after 3 h with an approximately 1.0 log reduction while a 4.0–5.0 log reduction after 48 h was observed in bile salt tolerance (Oh and Jung, 2015).

Cell surface hydrophobicity is one property to evaluate the ability of probiotic bacterial cells to adhere with colon mucosa. Probiotic bacteria must survive in the gastrointestinal tract condition and adhere to colon mucosa where probiotics have protective effects against pathogens through barrier functions (Monteagudo-Mera et al., 2019). In our investigation, the strains were thus tested for cell surface hydrophobicity challenged with hydrocarbons. As a result, all strains, i.e., BP8, BP156, and BP160 showed cell surface hydrophobicity of over 60% when challenged with hexadecane, toluene, and xylene. These findings were in line with the reports that probiotic strains of *L. rhamnosus* exhibited > 50% of hydrophobicity when challenged with xylene (Bhagat et al., 2020). from the human milk source, It was also found that probiotic strain *L. rhamnosus* exhibited good ability of cell surface hydrophobicity (33–69%) (Rajoka et al., 2017).

An adherence ability of probiotic bacteria carried out with the same probiotic species and pathogenic strains was evaluated through their auto-aggregation ability and co-aggregation ability, respectively. In this study, BP8, BP156, and BP160 strains had high auto-aggregation ability. Also, their high co-aggregation ability was found with *E. coli* (19.14–20.07%) and *S.* Enteritidis (20.82–21.66%) challenges, showing that BP8, BP156, and BP160 strains had high efficacy to prevent colonization of pathogens in the gastrointestinal tract. A previous study reported that probiotic strains obtained from human colostrum had 14.4–20.9% auto-aggregation (Krausova et al., 2019). It was reported that probiotic strain *P*. *pentosaceus* had rates of co-aggregation of 11.78 and 9.98% when challenged with *S*. *aureus* and *E*. *coli*, respectively (Taheur et al., 2016).

All considered aspects on probiotic criteria from the BP8, BP156, and BP160 strains were investigated including antimicrobial activity, non-hemolytic property, and survival in acid and bile salt conditions. Also, these strains were potential probiotics because they showed high abilities on cell surface hydrophobicity, auto-aggregation, and co-aggregation. Then, the genera identification by 16S rRNA sequencing and comparison revealed that these probiotics were defined as *Streptococcus salivarius* BP8, *S. salivarius* BP156, and *S. salivarius* BP160.

Application of these potential probiotics to impede liver and breast cancers was investigated in this study. The high antiproliferative effect of these *S. salivarius* strains on liver and breast cancer cells were revealed, implying that three probiotic strains *S. salivarius* BP8, *S. salivarius* BP156, and *S. salivarius* BP160 have anticancer potential against both liver and breast cancers. Similarly, antiproliferation of probiotic bacteria on MCF-7 and HepG2 cells has been reported (Luang-In et al., 2020). It has been reported that CFS of probiotic strain *Pediococcus* sp. is effective against MCF-7 cancer cells (Jafari-Nasab et al., 2021). In line with our study, the antiproliferative effect of a probiotic strain isolated from human breast milk against MCF-7 cancer cells was also reported (Jiang et al., 2016). Some metabolites produced from probiotic bacterial cells are widely mentioned due to their anticancer effect. As such, SCFAs are organic acid produced by probiotic bacteria that be considered as the substance to induce programmed cell death of cancer cells (Pizer et al., 1996). Thirabunyanon and Hongwittayakorn (2013) reported the SCFA bioproduction of probiotics and its effect on colon cancer cell antiproliferation.

DNA fragmentation was also investigated for apoptotic induction on cancer cells. As a result, small and very small DNA fragments were found on both liver and breast cancer cells treated with probiotic strains for 24 and 48 h, respectively. Our findings indicated that probiotic strains of *S. salivarius* BP8, *S. salivarius* BP156, and *S. salivarius* BP160 could induce apoptosis in liver and breast cancer cells via DNA fragmentation. A similar report was recorded wherein an apoptotic induction of colon cancer cells by DNA fragmentation was induced by probiotic strains (Dubey et al., 2016).

Based on morphological change evaluation after probiotic treatment for cancer cells prior to AO/PI staining, an apoptosis induction on liver and breast cancer cells was clearly found. Results indicated that all probiotic strains of *S. salivarius* BP8, *S. salivarius* BP156, and *S. salivarius* BP160 could induce apoptosis on liver and breast cancer cells showing an orange-stained cell appearance that indicated dead cells or late apoptosis. Similarly, a probiotic strain obtained from human breast milk was able to induce apoptotic programmed cell death on various human cancer cells (Nami et al., 2014).

A beneficial antioxidant substance was found to be a health promoter in humans and provides protection against several diseases in humans (Temple, 2000). In this investigation, antioxidative activity of *S. salivarius* BP8, *S. salivarius* BP156, and *S. salivarius* BP160 was elucidated. All forms of these probiotics including CFS, intact cells, and heat-killed cells were assessed, indicating the high constancy of probiotic *S. salivarius* BP160 strains. Likewise, an antioxidant activity of probiotic *Bacillus* spp. had DPPH scavenging activity of 2.57–27.34% (Ragul et al., 2020). Also, some reports mentioned that intact cells of some probiotic strains expressed more DPPH scavenging activity than CFS (Shivangi et al., 2020).

Protection against the free radicals of oxidative stress-induced cells was highly activated by *S*. *salivarius* CP8, *S. salivarius* CP156, and *S. salivarius* CP160 probiotic strains compared to the antioxidant compound of L-ascorbic acid. In fact, oxidative stress causes damage to biomolecules and proteins of the cells which is remarkably implicated with aging and human diseases (Estévez and Luna, 2017). Similarly, the protective capability of probiotics to detoxify hydrogen peroxide against oxidative stress-induced cells was elucidated (Coda et al., 2012; Arcanjo et al., 2019). Results indicated that these *S. salivarius* probiotic strains protect the cell against oxidative stress which is the primary cause of human diseases such as cancers.

## Conclusion

In this investigation of LAB derived from human breast milk, bacterial strains were analyzed based on probiotic characteristics including antimicrobial activity, non-hemolytic activity to red blood cells, survival in a simulated gastrointestinal tract, cell hydrophobicity, and aggregation ability. Sequencing by 16S rRNA, assessment, and comparison to GenBank revealed that these probiotics were *S. salivarius* BP8, *S. salivarius* BP156, and *S. salivarius* BP160. Proliferative inhibition of liver and breast cancer cells by these probiotic strains was observed. The mechanism of action of these probiotics was evaluated with apoptotic induction of DNA fragmentation and morphological change of AO/PI-stained cells. Moreover, their antioxidant and protective properties against cell oxidative stress activities were also highly activated. Thus, these probiotic strains of *S. salivarius* are suitable for application as potential probiotics for human health improvement and human cancer therapeutics.

## Data Availability Statement

The original contributions presented in the study are included in the article/supplementary material, further inquiries can be directed to the corresponding author/s.

## Author Contributions

MT contributed to conception and design of the study. KS performed the statistical analysis. KS and MT wrote the first draft of the manuscript. All authors contributed to manuscript revision, read, and approved the submitted version and organized the database.

## Conflict of Interest

The authors declare that the research was conducted in the absence of any commercial or financial relationships that could be construed as a potential conflict of interest.

## Publisher’s Note

All claims expressed in this article are solely those of the authors and do not necessarily represent those of their affiliated organizations, or those of the publisher, the editors and the reviewers. Any product that may be evaluated in this article, or claim that may be made by its manufacturer, is not guaranteed or endorsed by the publisher.
